# Barriers to initiation of hepatitis C virus therapy in Germany: A retrospective, case-controlled study

**DOI:** 10.1371/journal.pone.0250833

**Published:** 2021-05-10

**Authors:** Peter Buggisch, Hans Heiken, Stefan Mauss, Bernd Weber, Maria-Christina Jung, Herbert Görne, Renate Heyne, Holger Hinrichsen, Dennis Hidde, Bettina König, Ana Gabriela Pires dos Santos, Claus Niederau, Thomas Berg

**Affiliations:** 1 ifi-Institute for Interdisciplinary Medicine, Hamburg, Germany; 2 Private Practice, Hanover, Germany; 3 Center for HIV and Hepatogastroenterology, Düsseldorf, Germany; 4 Praxiszentrum Friedrichsplatz / Competence Center Addiction, Kassel, Germany; 5 Liver Centre, Munich, Germany; 6 MediZentrum Hamburg, Praxis für Suchtmedizin, Hamburg, Germany; 7 Leberzentrum am Checkpoint, Berlin, Germany; 8 Centre for Gastroenterology and Hepatology, Kiel, Germany; 9 AbbVie Deutschland GmbH & Co. KG, Wiesbaden, Germany; 10 AbbVie Inc., North Chicago, Illinois, United States of America; 11 Katholisches Klinikum Oberhausen, St. Josef-Hospital, Klinik für Innere Medizin, Akademisches Lehrkrankenhaus der Universität Duisburg-Essen, Oberhausen, Germany; 12 Division of Hepatology, Department of Medicine II, Leipzig University Medical Center, Leipzig, Germany; National Taiwan University Hospital, TAIWAN

## Abstract

Despite the availability of highly effective and well-tolerated direct-acting antivirals, not all patients with chronic hepatitis C virus infection receive treatment. This retrospective, multi-centre, noninterventional, case-control study identified patients with chronic hepatitis C virus infection initiating (control) or not initiating (case) treatment at 43 sites in Germany from September 2017 to June 2018. It aimed to compare characteristics of the two patient populations and to identify factors involved in patient/physician decision to initiate/not initiate chronic hepatitis C virus treatment, with a particular focus on historical barriers. Overall, 793 patients were identified: 573 (72%) who received treatment and 220 (28%) who did not. In 42% of patients, the reason for not initiating treatment was patient wish, particularly due to fear of treatment (17%) or adverse events (13%). Other frequently observed reasons for not initiating treatment were in accordance with known historical barriers for physicians to initiate therapy, including perceived or expected lack of compliance (14.5%), high patient age (10.9%), comorbidities (15.0%), alcohol abuse (9.1%), hard drug use (7.7%), and opioid substitution therapy (4.5%). Patient wish against therapy was also a frequently reported reason for not initiating treatment in the postponed (35.2%) and not planned (47.0%) subgroups; of note, known historical factors were also common reasons for postponing treatment. Real-world and clinical trial evidence is accumulating, which suggests that such historical barriers do not negatively impact treatment effectiveness. Improved education is key to facilitate progress towards the World Health Organization target of eliminating viral hepatitis as a major public health threat by 2030.

## Introduction

In recent years, the treatment landscape for chronic hepatitis C (CHC) virus infection has been dramatically improved with the advent of all-oral, interferon-free, direct-acting antiviral (DAA) therapies [1, 2]. Since 2016, three pangenotypic DAA regimens–sofosbuvir/velpatasvir, sofosbuvir/velpatasvir/voxilaprevir, and glecaprevir/pibrentasvir (G/P)–have been approved for the treatment of CHC by the European Medicines Agency [3–5]. These regimens achieve high rates of sustained virologic response (>95%) with good safety profiles [2, 6–12], and as such are recommended by international guidelines [13, 14].

Effective treatment with DAAs can reduce the risk of complications of CHC, including hepatocellular carcinoma, hepatic decompensation, and liver transplantation [15–17]. Furthermore, DAAs can be prescribed by a wide range of health care professionals (HCPs) experienced in treating patients with hepatitis C virus (HCV), including general practitioners and specialists across different relevant disciplines (eg, hepatologists, infectious disease experts, and addiction specialists).

Despite these advances, 71 million people globally are chronically infected with HCV, up to 80% of whom remain undiagnosed [18]. In 2016, the World Health Organization (WHO) adopted their “Global Health Sector Strategy on Viral Hepatitis, 2016‒2021,” setting a target of eliminating viral hepatitis as a major public health threat by 2030 by reducing new chronic infections by 90% and mortality from CHC by 65% [19]. However, few countries are on target to meet WHO elimination targets by 2030 [20].

Germany has one of the largest number of CHC cases in Europe commensurate with its large population size [1, 21]. Most cases are reported in injecting drug users (with a high HCV prevalence in new injectors), human immunodeficiency virus (HIV) coinfected men who have sex with men, and migrants from high HCV endemicity regions [22, 23]. An integrated strategy for HIV, hepatitis B and C, and other sexually transmitted infections was published in 2016 [8]. Before the COVID-19 pandemic and its disruption to the access to health care, Germany was on track to meet the WHO elimination target of 2030 [11], likely as a result of no restrictions on access to CHC treatment for diagnosed patients and universal reimbursement [9, 23].

Globally, despite the availability of effective therapies, many patients diagnosed with CHC remain untreated [24]. It is therefore important to investigate the reasons for a lack of treatment initiation in a real-world setting, from both the patient perspective and physician perspective, who may view historical barriers as reasons not to start or to postpone therapy (eg, comorbidities, older age, and drug use) [25]. The first CURRENT-C study conducted in Germany in 2014 provided some insight, demonstrating that patient choice was the most cited reason for not initiating CHC therapy, although this was conducted in the interferon/early DAA era [25].

Here, we present the results from a study investigating the baseline characteristics of patients diagnosed with CHC who received treatment compared with those who did not in the current era of DAAs. We identified reasons for physicians not initiating CHC treatment, including for patients with postponed treatment or no treatment planned at all. In particular, our focus was on physician-based historical barriers, with the reasons identified in the first CURRENT-C study used to inform this investigation.

## Methods

### Study design

This was a retrospective, noninterventional, case-control study involving patients attending 43 medical centres experienced in the treatment of CHC infection in Germany. The study was conducted in compliance with local ethics committee approval, local laws and regulations, and The Guidelines for Good Pharmacoepidemiology Practices in noninterventional studies. The study protocol was approved by the Ethics Committee of Ärztekammer Westfalen-Lippe (reference number 2014-395-f-S). All participants gave written informed consent prior to study inclusion.

The overall study duration was at least 20 months and consisted of a 10-month retrospective observation period followed by a 10-month documentation period. During the retrospective observation period (1 September 2017–30 June 2018), patients attended the medical centre at least once and a treatment decision was made. These patients were then enrolled in the study during a 10-month documentation period: 9 months for patient enrolment and retrospective data collection (1 July 2018–31 March 2019) followed by a month for data correction (April 2019). The final day of data collection was 31 March 2019.

### Study participants

Patient data were either collected as part of the CURRENT-C 2.0 (CC 2.0) study or from the German Hepatitis C registry (DHC-R). This study included all patients with CHC (HCV–ribonucleic acid positive for at least 6 months) aged 18 years or older at the start of the documentation period who consulted their HCP during the observation period at least once. Patients were excluded if they had acute HCV infection or had received treatment for CHC infection within 3 months before the start of the planned observation period.

Cases comprised patients for whom a decision not to treat the CHC infection was made by either the HCP or patient; controls were patients for whom a decision was made by HCP in agreement with the patient to start treatment. Cohorts of up to four controls for each case were selected by simple random sampling. For sites enrolling treated patients in the DHC-R, controls were selected with data from the German registry.

### Study endpoints and data collection

The primary objective of this study was to assess reasons for not initiating therapy in patients with CHC infection in Germany. Secondary objectives were to analyse patient and disease characteristics of untreated patients with CHC infection, document the proportion of patients with CHC infection who did not receive treatment, and evaluate decisions not to initiate treatment by type of study site.

Patient chart review data were collected from each site via electronic case report forms. Where patients had multiple visits to the HCP during the observation period, data from the final visit were analysed. Patient baseline characteristics were evaluated and categorised according to whether or not treatment was started. This included patient demographics, vital signs, disease characteristics, risk factors, treatment history, comorbidities, and comedications.

Reasons behind treatment decisions for CHC infection were collected. When opting for no immediate start of treatment for CHC infection, physicians were asked to complete a structured, multiple-choice questionnaire, in which reasons for not treating were determined a priori. The questionnaire presented a number of possible categories (eg, comorbidity) and subreasons (eg, coronary heart disease, psychiatric disease, etc.) behind the choice. Multiple categories and subreasons could be selected and there was no possibility to include any additional response.

Study site data were collected via a site survey. This survey included site-specific characteristics such as location and specialisation, the annual and total number of patients with CHC infection, type of setting (ie, hospital- versus office-based clinic), and annual number of patients with CHC infection who were treated.

To limit potential selection bias of the study sites not being representative of all HCP practices in Germany, study site selection covered a range of national regions as well as smaller and larger sites according to patient numbers, together with private practices and academic sites. The ratio of case to controls was selected to counter the potential limitation of the case-control study design.

### Statistical analysis

It was expected that approximately 5–10% of patients with CHC infection would not receive treatment. Therefore, the study planned to enrol a maximum of 1,000 patients with CHC infection (not exceeding 800 controls, and 200 cases) in order to detect an association between any predictive factor and the therapy decision. A Chi-square test was used for sample size calculations based on a power of 80% at a significance level of 5%, with an odds ratio of 2. Missing data were excluded from analyses. Subgroups analysed were patients with postponed treatment or had no treatment planned among those who did not receive therapy.

Case-control data were analysed descriptively using counts and percentages for categorical variables. In a first step, linear univariate regression analyses (binary, “treated” versus “untreated”) were performed to identify possible associations between patient characteristic and treatment initiation. These included the following parameters: sex, age, genotype 1a, genotype 1b, alcohol use, smoker status, cannabis use, injecting drug use, other illicit drug use, opioid substitution therapy (OST), cirrhosis status, infection with HCV via drugs, treatment history, employment status, heavy alcohol use (>40 g/d for males, >30 g/d for females), HIV coinfection, and psychiatric comorbidity. Multivariate logistics regression analyses with backward elimination were performed to assess the reasons for not initiating therapy, and only included significant parameters from the univariate linear regression analyses for which a documentation status with >75% valid information was available. The F-test for the multivariate regression analyses were set up with a minimum significance level of F as entry criterion (significance level 0.05) and a maximum significance level of F as an exclusion criterion for the elimination of a variable (significance level 0.1); up to 20 iterations were calculated.

## Results

### Characteristics of patients who did or did not receive treatment

In the participating centres, during the observation period, a total of 2,760 patients received HCV DAA therapy and 2,150 patients under care did not receive HCV DAA therapy. Of these, a total of 793 patients were included in the study; 394 (49.7%) and 399 (50.3%) from CC 2.0 and DHC-R, respectively. A total of 573 (72.3%; CC 2.0: n = 174, DHC-R: n = 399) patients received treatment and 220 (27.7%; CC 2.0: n = 220, DHC-R: n = 0) did not. Similar proportions of patients not initiating treatment were observed in hospital-based settings (20/95, 21%) and in office-based clinics (200/698, 29%). In total, 513 patients (64.7%) were male, 639 (80.6%) were treatment-naïve, 116 (14.6%) had compensated cirrhosis, and 2 patients (0.3%) had decompensated cirrhosis. In 25 (3.2%) patients, the cirrhosis status was not documented. The mode of infection was reported for 567 patients, of whom 65.4% were infected via drug use (intravenous/nasal); and 14.5%, 8.5%, 8.5%, and 3.2% via blood products, sexual transmission, surgical or medical intervention, and other routes, respectively.

**Fig 1:**
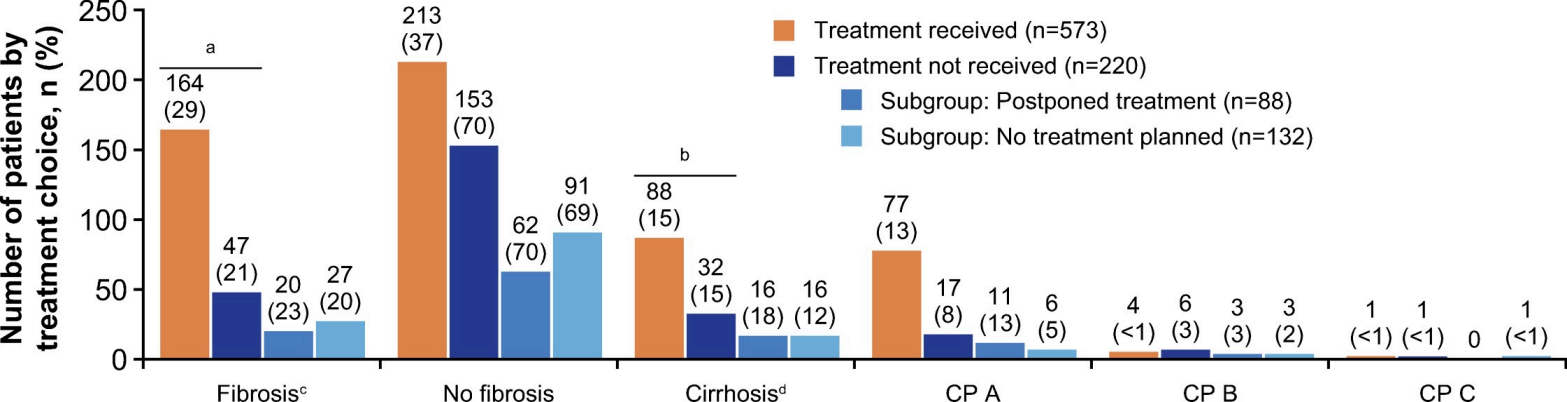
Fibrosis and cirrhosis status in patients with CHC virus infection. ^a^*P* = 0.001; ^b^*P* = 0.925 (received treatment vs treatment not received, two-sided Chi-square test); ^c^Fibrosis status was unknown in 6–196 (7–34%) of patients; ^d^CP status was unknown in 2–8 (0.7–5%) of patients with cirrhosis. CHC, chronic hepatitis C virus; CP, Child-Pugh.

Descriptive comparisons of the proportion of patients showed that a significantly higher proportion of untreated versus treated patients were heavy alcohol users (18.2% vs 12.2% respectively; *P* = 0.001). Significantly higher proportions of patients who did not receive treatment compared with those who did receive treatment had a history of cannabis use *(P* = 0.002) and injecting drug use (P <0.001) and were on OST (*P* <0.001), were older (<40 years or 40–60 years vs >60 years; *P* < 0.001) and of female gender (*P* = 0.001), while significantly fewer patients who did not receive treatment were infected with genotype 3 HCV versus all other genotypes (*P* = 0.046) (Table 1). Furthermore, significantly more patients who did not receive therapy compared with those who did receive therapy had any comorbidity (*P* <0.001), including cardiovascular disease (*P* = 0.001), psychiatric disease (*P* = 0.001) and HIV coinfection (*P* <0.001) (Table 2). No difference in the rate of cirrhosis (*P* = 0.925) was observed between the two groups, but significantly more patients who received treatment had fibrosis (*P* = 0.001) (Fig 1).

**Table 1:** Clinical characteristics and demographics in patients with CHC infection.

Characteristic	Patients	Patients not initiating treatment		
	receiving treatment	Overall	Patients	Patients with
		(n = 220)	with postponed treatment (n = 88)	no treatment planned (n = 132)
	(n = 573)			
**Male sex**	391 (68.2)	122 (55.5)^a^	56 (63.6)	66 (50.0)
**Age, y, median (range)**	50.0 (18–87)	51.5 (21–95)	47.0 (21–79)	56.0 (25–95)
** <40**	150 (26.2)	47 (21.4)	26 (29.5)	21 (15.9)
** 40–60**	321 (56.0)	97 (44.1)	46 (52.3)	51 (38.6)
** >60**	102 (17.8)	76 (34.5)^a^	16 (18.2)	60 (45.5)
**HCV genotype**^**b**^^,^^**c**^^,^^**d**^				
** 1**	328 (57.2)	130 (59.1)	42 (47.7)	88 (66.7)
** 1a**	189 (33.0)	57 (25.9)	24 (27.3)	33 (25.0)
** 1b**	131 (22.9)	65 (29.5)	14 (15.9)	51 (38.6)
** 2**	26 (4.5)	10 (4.5)	4 (4.5)	6 (4.5)
** 3**	185 (32.3)	55 (25.0)^a^	27 (30.7)	28 (21.2)
** 4**	26 (4.5)	11 (5.0)	7 (8.0)	4 (3.0)
** 5**	1 (0.2)	1 (0.5)	0 (0)	1 (0.8)
** 6**	0 (0.0)	2 (0.9)	1 (1.1)	1 (0.8)
**HIV coinfection**	40 (7.0)	39 (17.7)^a^	18 (20.5)	21 (15.9)
**HCV treatment-naïve**	458 (79.9)	181 (82.3)	72 (81.8)	109 (82.6)
**Cirrhotic**	86 (15.0)	32 (14.5)	16 (18.2)	16 (12.1)
**Non-cirrhotic**	471 (82.2)	179 (81.4)	68 (77.3)	111 (84.1)
**Alcohol intake,**	70 (12.2)	40 (18.2)^a^	24 (27.3)	16 (12.1)
**>40 g/day (men) or**				
**>30 g/day (women)**				
**History of cannabis use**	53 (9.2)	37 (16.8)^a^	22 (25.0)	15 (11.4)
**History of injecting**	45 (7.9)	48 (21.8)^a^	31 (35.2)	17 (12.9)
**drug use**				
**Opioid substitution therapy**	166 (29.0)	93 (42.3)^a^	50 (56.8)	43 (32.6)
**Any comorbidity**	438 (76.4)	199 (90.5)^a^	79 (89.8)	120 (90.9)
**Platelets**^**e**^**, μL, median (range)**	207,000	209,000	205,000	219,000
	(34,000–1,570,000)	(20,000–467,000)	(30,000–453,000)	(20,000–467,000)
**Employed**				
** Yes**	215 (37.5)	48 (21.8)	25 (28.4)	23 (17.4)
** No**	247 (43.1)	143 (65.0)^a^	49 (55.7)	94 (71.2)
** Unknown**	111 (19.4)	29 (13.2)	14 (15.9)	15 (11.4)
**Educational level**				
** Secondary school certificate (Hauptschule)**	104 (18.2)	43 (19.5)	26 (29.5)	17 (12.9)
** Secondary school certificate (Realschule)**	91 (15.9)	22 (10.0)	11 (12.5)	11 (8.3)
** High school graduation certificate (Abitur)**	28 (4.9)	10 (4.5)	4 (4.5)	6 (4.5)
** University degree**	22 (3.8)	5 (2.3)	3 (3.4)	2 (1.5)
** Unknown**	328 (57.2)	140 (63.6)	44 (50.0)	96 (72.7)

Data are n (%) unless otherwise stated.

^a^*P* <0.05.

^b^*P* = 0.127 (received treatment vs treatment not received, two-sided Chi-square test).

^c^Treatment received, n = 570; treatment not received, n = 209; treatment postponed, n = 81; no treatment planned, n = 128.

^d^Percentages refer to total group.

^e^Treatment received, n = 532; treatment not received, n = 206; treatment postponed, n = 81; no treatment planned n = 125.

CHC, chronic hepatitis C virus; HCV, hepatitis C virus; HIV, human immunodeficiency virus.

**Table 2:** Comorbidities in patients with CHC infection.

Comorbidity^a^	Patients	Patients not initiating treatment		
	receiving treatment	Overall	Patients	Patients with
		(n = 220)	with postponed treatment	no treatment planned
	(n = 573)			
			(n = 88)	(n = 132)
**Diabetes mellitus**	39 (6.8)	19 (8.6)	5 (5.7)	14 (10.6)
** Type I**	4 (0.7)	2 (0.9)	0 (0.0)	2 (1.5)
** Type II**	35 (6.1)	17 (7.7)	5 (5.7)	12 (9.1)
**Skin disorder**	7 (1.2)	15 (6.8)	5 (5.7)	10 (7.6)
**History of any drug abuse**	220 (38.4)	101 (45.9)^b^	53 (60.2)	48 (36.4)
** Current**	25 (4.4)	33 (15.0)	16 (18.2)	17 (12.9)
** Former**	80 (14.0)	20 (9.1)	13 (14.8)	7 (5.3)
**Opioid substitution therapy**	166 (29.0)	93 (42.3)^c^	50 (56.8)	43 (32.6)
**Cardiovascular disease**	95 (16.6)	59 (26.8)^c^	12 (13.6)	47 (35.6)
** Coronary heart disease/angina pectoris/myocardial infarction**	12 (2.1)	22 (10.0)	3 (3.4)	19 (14.4)
**Other**	89 (15.5%)	49 (22.3)	9 (10.2)	40 (30.3)
**Thyroid disorder**	42 (7.3)	14 (6.4)	4 (4.5)	10 (7.6)
**Psychiatric disease**	88 (15.4)	56 (25.5)^c^	29 (33.0)	27 (20.5)
** Depression**	66 (11.5)	31 (14.1)	15 (17.0)	16 (12.1)
** Psychosis**	15 (2.6)	8 (3.6)	6 (6.8)	2 (1.5)
** Other psychatric disease**	20 (3.5)	27 (12.3)	14 (15.9)	13 (9.8)
**Chronic kidney disease**	19 (3.3)	12 (5.5)	5 (5.7)	7 (5.3)
** Dialysis**	10 (1.7)	12 (5.5)	5 (5.7)	7 (5.3)
** No dialysis**	9 (1.6)	0 (0.0)	0 (0.0)	0 (0.0)
** Malignant disease**	13 (2.3)	17 (7.7)	3 (3.4)	14 (10.6)
** Non-liver related**	11 (1.9)	14 (6.4)	2 (2.3)	12 (9.1)
** Liver related**	6 (1.0)	4 (1.8)	1 (1.1)	3 (2.3)
**HIV coinfection**	40 (7.0)	39 (17.7)^c^	18 (20.5)	21 (15.9)
**Hepatitis B virus coinfection**	16 (2.8)	10 (4.5)	2 (2.3)	8 (6.1)
**Neurological disorder**	15 (2.6)	9 (4.1)	2 (2.3)	7 (5.3)
** Post stroke**	1 (0.2)	5 (2.3)	0 (0.0)	5 (3.8)
**Other comorbidity**	245 (42.8)	85 (38.6)	21 (23.9)	64 (48.5)

All data are n (%).

^a^Multiple comorbidities of the same overall group could be selected.

^b^*P* = 0.054.

^c^*P* <0.05 (received treatment vs treatment not received, two-sided Chi-square test).

CHC, chronic hepatitis C virus; HIV, human immunodeficiency virus.

### Reasons against initiating treatment

#### Patient perspective

Patient wish against therapy was the most frequently reported patient’s reason for not initiating treatment (42.3%), particularly due to fear of treatment (17.3%) or fear of adverse events (13.2%) (Table 3). However, patient wish was a reason in significantly fewer people who inject drugs compared with those who do not (29.2% [14/48] vs 50.0% [64/128]; *P* = 0.013), and in numerically fewer patients on OST (35.5% [33/93] vs 47.2% [60/127] respectively; *P* = 0.081).

**Table 3:** Reasons reported for not initiating CHC treatment (>4% of patients).

Reason^a^	Patients not initiating treatment		
	Overall	Patients	Patients with
		with postponed treatment	no treatment planned
	(n = 220)	(n = 88)	(n = 132)
**Patient wish**^**a**^	93 (42.3)	31 (35.2)	62 (47.0)^b^
** Fear of treatment**^**c**^	38 (17.3)	11 (12.5)	27 (20.5)
** Fear of adverse events**	29 (13.2)	13 (14.8)	16 (12.1)
** Lack of illness insight/acceptance**	25 (11.4)	4 (4.5)	21 (15.9)^d^
** Family- or job-related reasons**	17 (7.7)	11 (12.5)	6 (4.5)^d^
** Special personal reasons**	22 (10.0)	8 (9.1)	14 (10.6)
**Lack of compliance**	32 (14.5)	14 (15.9)	18 (13.6)
**High patient age**	24 (10.9)	0 (0.0)	24 (18.2)^d^
**Ongoing drug abuse**^**a**^^,^^**e**^	33 (15.0)	23 (26.1)	10 (7.6)^d^
** Alcohol abuse**	20 (9.1)	14 (15.9)	6 (4.5)^d^
** Hard drug abuse**^**f**^	17 (7.7)	11 (12.5)	6 (4.5)^d^
** Opioid substitution therapy**	10 (4.5)	8 (9.1)	2 (1.5)^d^
**Any comorbidity**^**g**^	33 (15.0)	10 (11.4)	23 (17.4)
** Psychiatric illness**	17 (7.7)	10 (11.4)	7 (5.3)
**Lack of disease progression**	13 (5.9)	3 (3.4)	10 (7.6)
**Other reasons**	24 (10.9)	7 (8.0)	17 (12.9)

All data are n (%)

^a^Multiple selections were allowed.

^b^*P* = 0.084.

^c^Fear of treatment consisted of intrinsic anxiety related to treatment, eg, loss of control, disruption of daily activities, etc.

^d^*P* <0.05 (postponed treatment vs no planned treatment, two-sided Chi-square test).

^e^Including alcohol abuse, hard drug abuse, soft drug abuse and opioid substitution therapy.

^f^Heroine, amphetamine, cocaine, designer drugs (or none of these selected).

^g^Excludes drug abuse, alcohol abuse and psychiatric disease.

CHC, chronic hepatitis C virus.

Patients aged ≥40 years expressed a wish against therapy significantly more often than patients aged <40 years because of fear of treatment (20.2% [35/173] vs 6.4% [3/47]; *P* = 0.03) or fear of side effects (16.2% [28/173] vs 2.1% [1/47]; *P* = 0.01). These reasons were also more common in patients with a longer compared with shorter duration of infection: the average duration of infection in patients not initiating treatment due to a lack of disease activity (26 years, n = 6), fear of adverse events (22 years, n = 18), fear of treatment (21 years, n = 19) or patient wish (20 years, n = 40) was, on average, numerically longer than that of the overall population (18 years, n = 106). The percentage of patients who expressed a wish against therapy was significantly higher in female patients (51.0% [50/98] than in male patients (35.2% [43/122]; *P* = 0.019).

#### Physician-based historical barriers

The other frequently observed reasons for not initiating treatment were in accordance with the known historical barriers for physicians not to start therapy, including perceived or expected lack of compliance (14.5%), high patient age (10.9%), comorbidities (15.0%), alcohol abuse (9.1%), hard drug abuse (7.7%) and OST (4.5%). In patients who were heavy alcohol users, 45.0% (18/40) did not receive therapy because of their alcohol abuse. Of 56 patients who had concomitant psychiatric disease, treatment was not initiated by the physician in 19 (33.9%) because of perceived or expected lack of compliance, compared with 13/164 (7.9%) of those without this comorbidity.

Historical physician-based barriers were also among significant predictors of not initiating treatment in univariate logistics regression analyses. These were high age (≤60 years vs >60 years; *P* < 0.001), HIV coinfection (*P* <0.001), psychiatric comorbidity (*P* = 0.001), cannabis use (*P* = 0.001), injecting drug use (*P* <0.001), other illicit drug use (*P* <0.001), and OST (*P* <0.001) (Table 4). Additional significant predictors of not initiating treatment were unemployment (*P* <0.001) and female sex (*P* = 0.001). Older age (*P* <0.001), female sex (*P* <0.001), HIV coinfection (*P* <0.001), injecting drug use (*P* = 0.001), and OST (*P* = 0.001) were still significant predictors of not initiating treatment in multivariate logistics regression analyses.

**Table 4:** Univariate and multivariate analyses of predictive factors for initiating CHC treatment.

	*P* value	OR	95% CI
Parameter			
**Univariate analysis**			
** Sex, male vs female**	0.001	1.726	1.255–2.374
** Age, ≤60 years vs >60 years**	<0.001	2.437	1.716–3.461
** Genotype 1b vs other genotypes**	0.051	0.707	0.498–1.002
** Genotype 1a vs other genotypes**	0.054	1.407	0.994–1.994
** Alcohol use, yes vs no**	0.902	0.978	0.691–1.386
** Smoker**			
** Current smoker vs nonsmoker**	0.658	0.922	0.643–1.321
** Current smoker vs ex-smoker**	0.848	1.071	0.534–2.147
** Cannabis use, yes vs no**	0.001	0.443	0.276–0.711
** Injecting drug use, yes vs no**	<0.001	0.234	0.149–0.368
** Other illicit drug use, yes vs no**	<0.001	0.264	0.161–0.432
** Opioid substitution therapy, yes vs no**	<0.001	0.557	0.403–0.769
** Cirrhosis, no vs yes**	0.925	1.021	0.657–1.587
** Possible mode of infection with HCV: drugs (IV or nasal), no vs yes**	0.261	1.195	0.876–1.632
** Treatment history, no vs yes**	0.456	0.926	0.758–1.133
** Employment, yes vs no**	<0.001	2.593	1.783–3.772
** Heavy alcohol use >40g/d (m), >30g/d (f), yes vs no**	0.01	1.786	1.148–2.777
** HIV coinfection, yes vs no**	<0.001	0.348	0.217–0.558
** Psychiatric comorbidity yes vs no**	0.001	0.531	0.364–0.776
**Multivariate analysis**^**a**^			
** Age, ≤60 years vs >60 years**	<0.001	6.258	3.586–10.921
** Sex, male vs female**	<0.001	2.450	1.534–3.912
** HIV coinfection, no vs yes**	<0.001	0.194	0.094–0.403
** Injecting drug use, yes vs no**	0.001	0.310	0.155–0.617
** Opioid substitution therapy, yes vs no**	0.001	0.373	0.205–0.678
** Other illicit drug use, yes vs no**	0.051	0.486	0.236–1.002
** Psychiatric comorbidity, no vs yes**	0.076	0.612	0.356–1.052

^a^Multivariate logistics regression analyses with backward elimination for significant parameters with at least 75% valid cases (n = 585).

CHC, chronic hepatitis C virus; CI, confidence interval; HCV, hepatitis C virus; IV, intravenous; OR, odds ratio.

**Fig 2:**
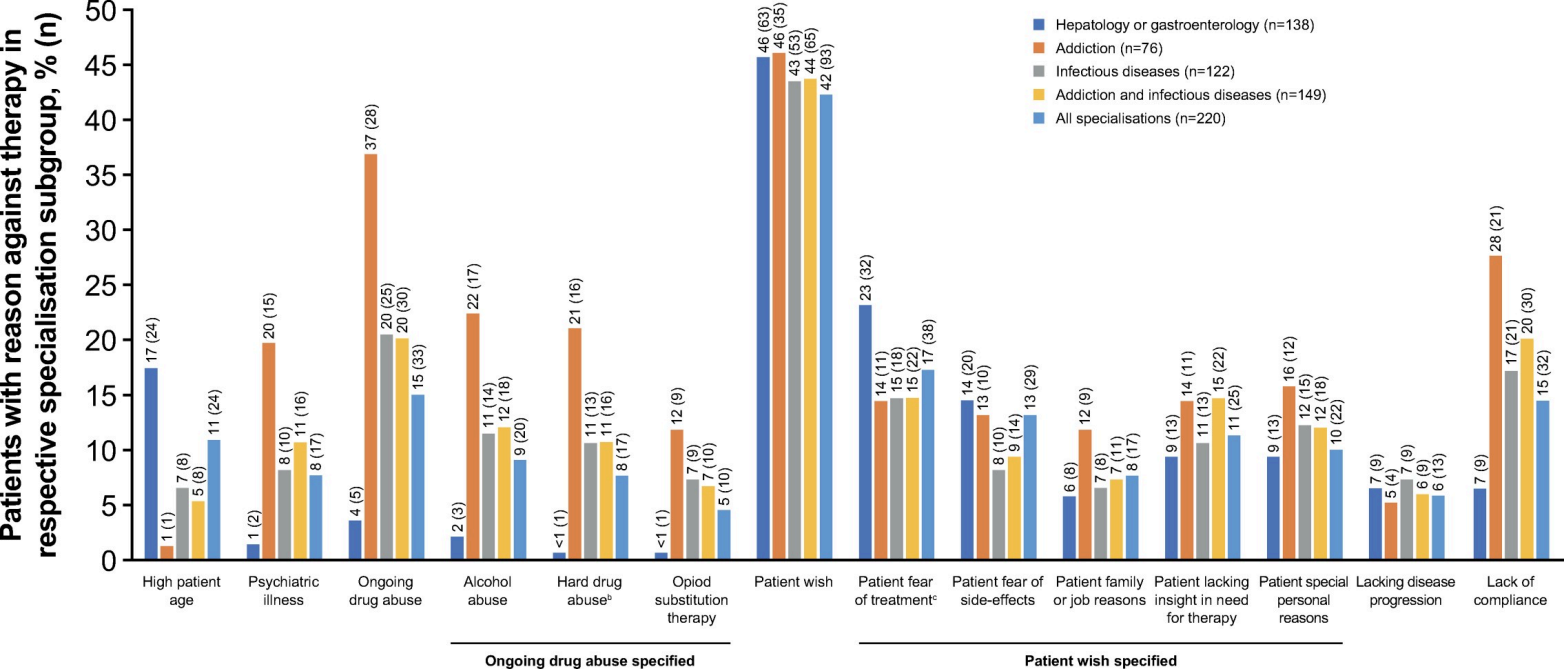
Reasons (>4% of patients) for not initiating treatment by physician’s medical specialisation^a^. ^a^As specified by site, multiple selections allowed; ^b^Heroine, amphetamine, cocaine, designer drugs (or none of these selected); ^c^Fear of treatment consisted of intrinsic anxiety related to treatment, eg, loss of control, disruption of daily activities, etc.

**Fig 3:**
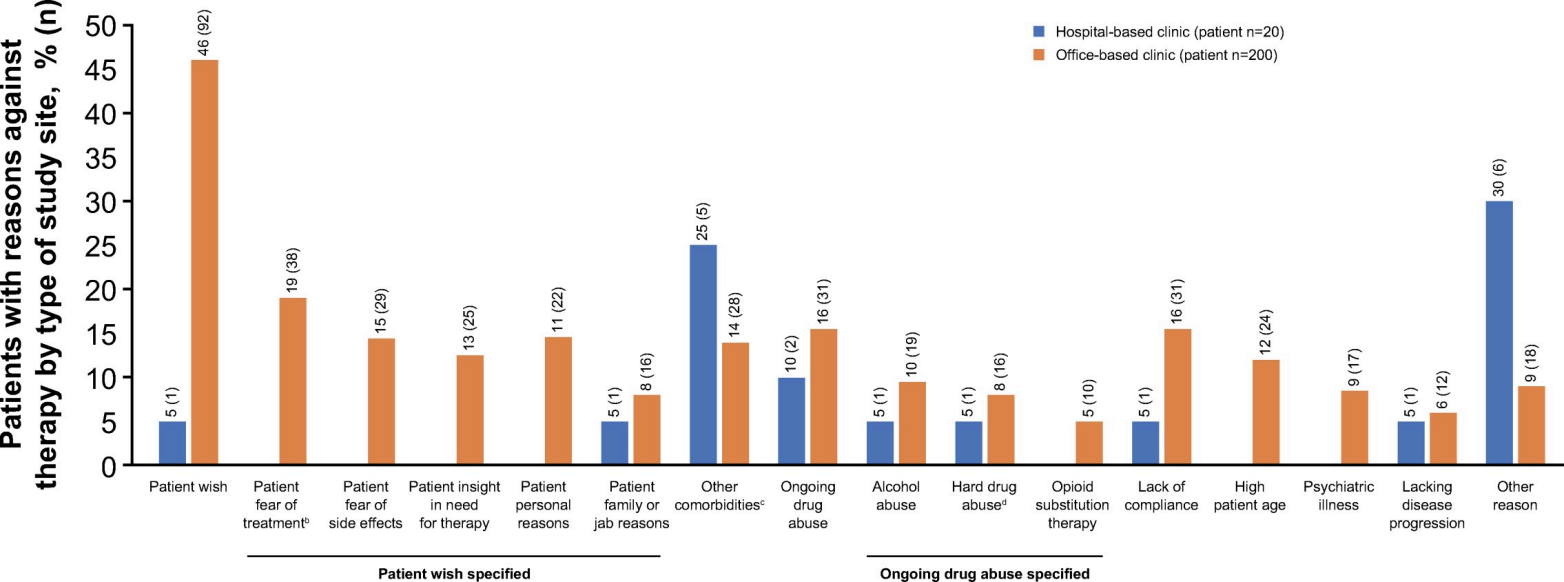
Reasons (>4% patients) for not initiating treatment by type of study site^a^. ^a^Multiple selections were allowed; ^b^Fear of treatment consisted of intrinsic anxiety related to treatment, eg, loss of control, disruption of daily activities, etc; ^c^Include decompensated disease, coronary heart disease, diabetes, skin disorders, immunological-inflammatory disorder, neurological disorder, and human immunodeficiency coinfection; ^d^Heroine, amphetamine, cocaine, designer drugs (or none of these selected).

Reasons for not initiating therapy were also analysed from the perspective of the physician’s medical specialisation (ie, hepatologists or gastroenterologists, infectious disease experts and addiction specialists) and type of clinical medical setting (ie, hospital- or office-based clinic). Fear of treatment or adverse events and patient age were the most common reasons for hepatologists or gastroenterologists to not initiate treatment, compared with the other specialties; use of hard drugs or continued drug use, alcohol abuse, OST, and poor compliance were also common for addiction specialists (Fig 2). Presence of other comorbidities was the most common reason for hospital-based physicians to not initiate treatment; patient’s preference (predominantly because of fear of treatment or adverse events), poor compliance, and continued drug abuse were the most common reasons reported in office-based clinics (Fig 3).

### Characteristics of patients with postponed treatment or had no treatment planned

Of those not initiating treatment, 88 (40.0%) postponed treatment and 132 (60.0%) had no treatment planned (Table 1). A greater proportion of patients in the no-planned-treatment group were aged 60 years and older compared with the postponed treatment group (aged <40 years or 40–60 years vs >60 years; *P* < 0.001) and were infected with genotype 1b (*P* <0.001). However, a greater proportion of patients with postponed treatment compared with no treatment planned were heavy alcohol users (*P* = 0.025), had a history of cannabis use (*P* = 0.034) and injecting drug use (*P* <0.001), and were on OST (*P* <0.001). No difference in the rate of fibrosis or cirrhosis was observed between the two subgroups (*P* = 0.938 and *P* = 0.201, respectively) (Fig 1).

### Reasons against initiating treatment

#### Patient perspective

Patient wish against therapy was the most frequently reported patient’s reason for not initiating treatment in the postponed (35.2%) and not planned (47.0%) subgroups, though the difference was not significant (*P* = 0.084) (Table 3). A significantly higher proportion of patients with no treatment planned had a lack of illness insight/acceptance compared with those with postponed treatment *(P* = 0.009).

#### Physician-based historical barriers

The proportion of patients with older age as a reason to not receive treatment was higher for no treatment planned compared with those with postponed treatment (*P* <0.001) (Table 3). Reasons that were significantly higher in the postponed treatment group were a history of alcohol abuse (*P* = 0.004), hard drug abuse (*P* = 0.030), or ongoing drug abuse including alcohol (*P* <0.001) and OST (*P* = 0.008).

## Discussion

In this real-world retrospective, noninterventional, case-control study, 27.7% of documented patients with CHC infection did not receive treatment. One of the main reasons for not initiating therapy was patient wish against treatment, particularly due to fear of treatment. However, known historical barriers for physicians to initiate therapy were also still common reasons for patients not to receive or to postpone treatment.

The retrospective design of this study with consecutive patient selection minimises the risk of selection bias [26]. Furthermore, the real-world setting utilising data from physicians of a range of specialties, in both private practices and academic sites, provides insight into the care cascade of patients diagnosed with CHC in clinical practice. The large number of patients included overall enhances the validity of the observations reported, albeit there were relatively low numbers of patients within the individual subgroups. Limitations of the study include the reliance on retrospectively analysed electronic patient data, which are not directly obtained from patients and may be inconsistently recorded or have missing data, a caveat of many real-world, retrospective studies. For example, further characterisation of whether active drug use affects barriers to treatment is likely to be of interest, but was not captured in this study. Furthermore, the primary objective of this study (ie, reasons for not initiating therapy) is subjective, thus inherently prone to bias. Finally, the limited follow-up duration of the study necessitates the need for further studies to characterise the patient journey related to future treatment decisions.

Despite the wide availability of effective DAAs to treat HCV, a substantial proportion of patients diagnosed with CHC infection are still not initiating indicated treatment [27–30]. Patient preference seems to play a key role in this lack of initiation in clinical practice. The original CURRENT-C study, conducted in the interferon/early DAA era [25], reported that 24% of patients diagnosed with CHC infection refused treatment, primarily for fear of treatment toxicity or for family or job reasons [25]. Of those patients delaying treatment to a later date, the vast majority (80%) were said to be waiting until the approval of broadly accessible interferon-free therapies [25]. However, our findings show that despite the availability of DAAs, many patients diagnosed with CHC still did not receive treatment. Furthermore, compared with the earlier CURRENT-C study, in a greater proportion of patients who did not receive therapy, the choice was made to not initiate therapy at all, rather than postponing to a later date [25]. In addition to this, the proportion of patients not initiating treatment as a result of patient preference in the present study is almost twice that reported in the earlier study [25]. Notably, the historical barriers to treatment initiation identified in the first CURRENT-C study (older age, drug use, and comorbidities), involving patients for whom clinical and real-world evidence now support use [31–36], were still evident in this investigation. Fear of treatment and adverse events were also important reasons for not initiating treatment, but given that DAAs are orally administered and have good safety profiles [2, 6–12], in clinical practice these are likely to be essentially the same reason.

Several other studies have also investigated potential barriers to HCV treatment. Concerns regarding adherence of patients to both treatment and care appointments are supported by other retrospective studies of patients with CHC that have shown as many as 30–67% of patients diagnosed with CHC and not initiating treatment did so as a result of poor adherence to clinic appointments or loss to follow-up [27, 37]. Similar to this study, others have also shown that advanced age [25], comorbidities (in particular psychiatric illness), as well as alcohol and drug abuse [37], can be associated with nontreatment in patients with CHC [27, 28]. One recent study detailing physician attitudes towards CHC treatment in patients receiving opioid agonist treatment (OAT) in the United States, cited the perceived need for stable alcohol use and OAT, concerns regarding adherence, and challenging and marginalised patient lives as potential barriers to CHC treatment in this population [29].

Despite differences between interferon-based and interferon-free therapy, a previous prospective cohort study of more than 13,000 patients and 434 physicians conducted in Germany during the interferon era (2003–2008) reported similar barriers to treatment with pegylated interferon-α2a/ribavirin as seen with DAAs [38]. Patient wish was the most common reason against initiating treatment (63%). Among these patients, lack of understanding of the need of therapy, family and job problems, and fear of side effects were frequently reported reasons. The latter was mentioned more often by women than men (30% vs 19%; *P* < 0.001). Alcohol or drug abuse and comorbid diseases (depression was the most commonly reported) were also frequent treatment barriers. Lack of liver disease, symptoms, and fibrosis, as well as normal alanine aminotransferase levels were reasons mentioned by patients who did not see the need for treatment.

This study, as well as a US study conducted in the interferon era, showed a lower uptake of treatment in women compared with men [38, 39], in agreement with the present analysis conducted in the DAA era. The sex factor was unexpected because men have been shown to have a lower use of medical services than women both in the United States [40] and Germany [41]. Therefore, good knowledge and care about health issues per se do not necessarily increase treatment uptake for HCV in the interferon era. Interestingly and unexpectedly, the same picture is still true in the era of DAA therapy that lacks almost all side-effects previously observed with interferon-based therapies. A possible explanation behind the low treatment uptake among women in this study might be explained by the higher median age of women versus men: 56.0 vs 47.5 years; mean (standard deviation) 59.4 (17.4) vs 50.8 (15.5) years, respectively, *P* = 0.0001. Data in this study show that older age is a significant predictor for not initiating therapy, where the most frequent reasons against therapy were wish against treatment and old patient age.

This study provides insight into which patients do not receive treatment of CHC infection and the reasons for noninitiation. Although geographically limited to patients in Germany, the results are likely to be relevant to CHC patients in other countries, including those with a similarly high HCV burden, such as Italy, France, and the United Kingdom [1], or those with similarly open access to DAAs, such as The Netherlands [9].

A key finding of this study is that known historical barriers (eg, perceived or expected lack of compliance, high patient age, HIV coinfection, psychiatric comorbidities, alcohol abuse, hard drug use, and OST) for physicians to initiate therapy remain despite the availability of highly effective DAAs. When examining reasons for not initiating treatment by type of clinical setting (ie, hospital- or office-based clinic) and/or speciality of the physician, known barriers seem to be consistent with the typical patient populations treated in each setting/speciality rather than different access to care. For example, patients treated in hospitals tend to have more comorbidities than those attending office-based clinics, and addiction specialists will care for patients with ongoing drug/alcohol abuse and psychiatric illness. Several historical factors, such as drug or alcohol abuse, are also associated with a greater likelihood that treatment will be postponed rather than not initiated at all. However, evidence from real-world registries, postmarketing observational studies, and clinical trials suggests that DAAs are highly effective in patients whose circumstances would have historically prevented or led to postponed DAA treatment. For example, recent real-world studies report that glecaprevir/pibrentasvir achieved sustained virologic response at posttreatment Week 12 (SVR12) rates of ≥93.4% even in the presence of a psychiatric disorder, drug use (active, recent, or former), OST, and alcohol dependence or abuse, with a rate of ≥90.9% reported, even in patients with lower than 90% treatment adherence [31, 34, 36]. High SVR12/24 rates were also observed in patients on OST treated with elbasvir/grazoprevir (98.4%) [33] and DAAs in general (96%) [32] in further data from the real-world DHC-R registry, and in patients on OAT in the CO-STAR clinical trial (96%) [35]. Similarly, in a Phase IV clinical trial, high SVR12 rates were observed in heavy drinkers treated with ledipasvir/sofosbuvir (93.3%) [42]. Given the effectiveness of DAA-treatment in such patient populations, there is a need to educate physicians that barriers that have historically prevented or led to postponed treatment should be removed.

Educating patients about the importance of CHC treatment is also crucial, particularly as decisions regarding treatment initiation may be based on false information. The current study has provided further insight into patient populations who should be targeted for education, and the concerns that need to be addressed (eg, fear of treatment). Such education has potential to encourage more patients to initiate CHC treatment and improve adherence to both treatment and clinic appointment schedules. Supporting this concept, a study in rural China showed that HCV education markedly improved acceptance of antiviral therapy [43]. Moreover, HCV education of drug users on methadone maintenance treatment significantly increased treatment interest (*P* <0.001) in a Malaysian study [44]. Education regarding the potential consequences of disease progression in the absence of treatment is also important, especially as a fear of treatment or side effects was associated with a longer disease duration in this study. The latter increases the risk of serious complications of CHC infection, such as hepatic decompensation or hepatocellular carcinoma [17, 45, 46]. In this real-world study, 28% of the documented patients with chronic HCV infection did not receive indicated CHC treatment, despite the availability of effective pangenotypic interferon-free DAAs. Nonmedical reasons, such fear of treatment, were frequently cited as the reason for treatment noninitiation. Whilst this study confirms that historical barriers to HCV treatment remain, evidence is accumulating that not initiating or postponing treatment in such patients is unwarranted because treatment is highly effective. Overcoming known historical barriers, as well as educating hesitant patients and encouraging physician–patient discussion regarding the importance of treatment, are imperative to drive efforts towards meeting the WHO target of eliminating viral hepatitis as a major public health threat by 2030 [19].
